# Evidence That Calls-Based and Mobility Networks Are Isomorphic

**DOI:** 10.1371/journal.pone.0145091

**Published:** 2015-12-29

**Authors:** Michele Coscia, Ricardo Hausmann

**Affiliations:** Center for International Development, Harvard University, Cambridge MA, United States of America; East China University of Science and Technology, CHINA

## Abstract

Social relations involve both face-to-face interaction as well as telecommunications. We can observe the geography of phone calls and of the mobility of cell phones in space. These two phenomena can be described as networks of connections between different points in space. We use a dataset that includes billions of phone calls made in Colombia during a six-month period. We draw the two networks and find that the call-based network resembles a higher order aggregation of the mobility network and that both are isomorphic except for a higher spatial decay coefficient of the mobility network relative to the call-based network: when we discount distance effects on the call connections with the same decay observed for mobility connections, the two networks are virtually indistinguishable.

## Introduction

Telecommunications was supposed to bring forth the death of distance in social relations. New technologies permit people to communicate instantaneously around the globe. Yet, many social and economic characteristics and behaviors remain strongly affected by distance, such as trade [1], investment [2], research and development, and knowledge spillovers. This literature has looked at the impact of distance on the patterns of patent citation [3], of R&D and patent output [4, 5], of R&D and productivity [6], and on the sales of subsidiaries of multinational corporations [7].

New kinds of data have allowed the study of human behavior in space. These methods include tracking the actual movements of people across the territory, by looking at how dollar bills travel across the US [8], by looking at the social connections they establish [9, 10], or by directly observing human mobility [11]. Previous research has shown that on-line social media [12] and Twitter [13] also are strongly affected by distance, a fact that has often been interpreted as a consequence of homophily, meaning that people tend to connect with people similar to themselves [14]. But homophily must be itself endogenous to some other forms of human interaction that are affected by distance. Predicting mobility and social ties has had many applications in computer science [15–18], especially using temporal patterns [19]. Applications include, for instance, the impact of social influence on usage of city areas [20, 21], ride sharing services [22, 23], the impact of epidemics [24–26] and the study of mobility motifs [27]. Researchers have been able to relate (and predict) relevant facts about the economy by looking at mobility and social media usage [28, 29].

In this paper we advance this research by studying the relationship between the call network as captured by cell phone usage and the mobility network as captured by cell phones movements. We observe more than two billion calls made by around seven million phone numbers in Colombia during a six-month period. We do not have access to any phone numbers or identity of the phone users, and no personal data has been shared by the telecommunication operators. Each phone number is encrypted and anonymized and we preserve the users’ privacy by aggregating our data at the municipal level. Each phone is associated with the municipality where it spends most of the time. We are then able to reconstruct the network of call relations across Colombian municipalities by looking at the phone usage between municipalities. At the same time, we are able to track the movements of each phone, since we know the cellphone tower with which it connects to initiate its calls. We use this data to determine the network of human flows across municipalities. Our aim is to study the relationship between these two networks. To do so, we group municipalities into coherent modules or clusters: municipalities in the same module have strong relations to each other and weak relations to municipalities outside the module. We then compare the calls-based modules with the mobility modules.

As in many other areas of human activity, we also find that call connections based on phone usage decay strongly with distance, even though the cost of the calls themselves are unaffected by distance. This means that the intensity of the underlying call interactions that make people call each other do decay with distance. We find that calls-based modules are geographically compact.

Our main finding in this paper is that the network captured by phone calls is a higher-order aggregation of human mobility. When we compare the calls-based modules with the mobility modules, we see that call modules are larger and include many neighboring mobility modules. Each mobility cluster can be assigned to a parent call cluster and very few municipalities escape this hierarchical order—a number that is an order of magnitude lower than random expectation. To the best of our knowledge the intimate and hierarchical relationship between the calls-based and the mobility networks is a new result. We push the result further by rescaling call connections as if they were influenced by distance to the same degree as mobility connections. When performing this operation, we observe a remarkable correlation between the rescaled call-based network and the mobility network. We present this observation as the first evidence that call-based and mobility networks are isomorphic, which is a stronger results than the one so far discussed in the literature, where the two types of networks are just considered correlated. This better understanding of the relationship between telecommunications-based and face-to-face social life could improve our current applications that rely on human mobility, for example geographical marketing studies [30], and the investigation of the ties between economic development and mobility [31].

Mobility clusters in Colombia have already been delineated and studied recently [32], using transportation-based commuting data and focusing exclusively on the most populous areas of Colombia. We use these results to test the robustness of our subdivision of the Colombian territory. Our results are in agreement with this independent study, implying that our data is capturing a robust pattern.

## Results

### Network Topology

From cellphone usage data we are able to create a calls-based social network *S* of Colombia. In this network we aggregate the data at the municipality level, connecting two municipalities if there is a significant number of calls between them. For details about the municipality aggregation, edge creation and significance threshold, see the Method section. A similar procedure is employed to build the mobility network *M*. In this case, too, we aggregate at the municipality level. Municipalities are connected if we observe a significant number of trips flowing from one municipality to the other. Both networks are asymmetric, i.e. the strength of the connection from municipality *m*_1_ to municipality *m*_2_ is not the same of the one from *m*_2_ to *m*_1_. This asymmetry is significant in both the mobility and in the calls-based network. In the mobility network we are tracking movements from one place to another. Thus, we expect to find popular places attracting visitors more than other places, in line with classical geographic theories like the central place theory [33]. In the call-based network, we also expect an asymmetry due to the nature of the data: some people are initiators of social relationships and some are social attractors. The asymmetric nature of some social relations has been studied multiple times in the literature [34–36].

**Fig 1:**
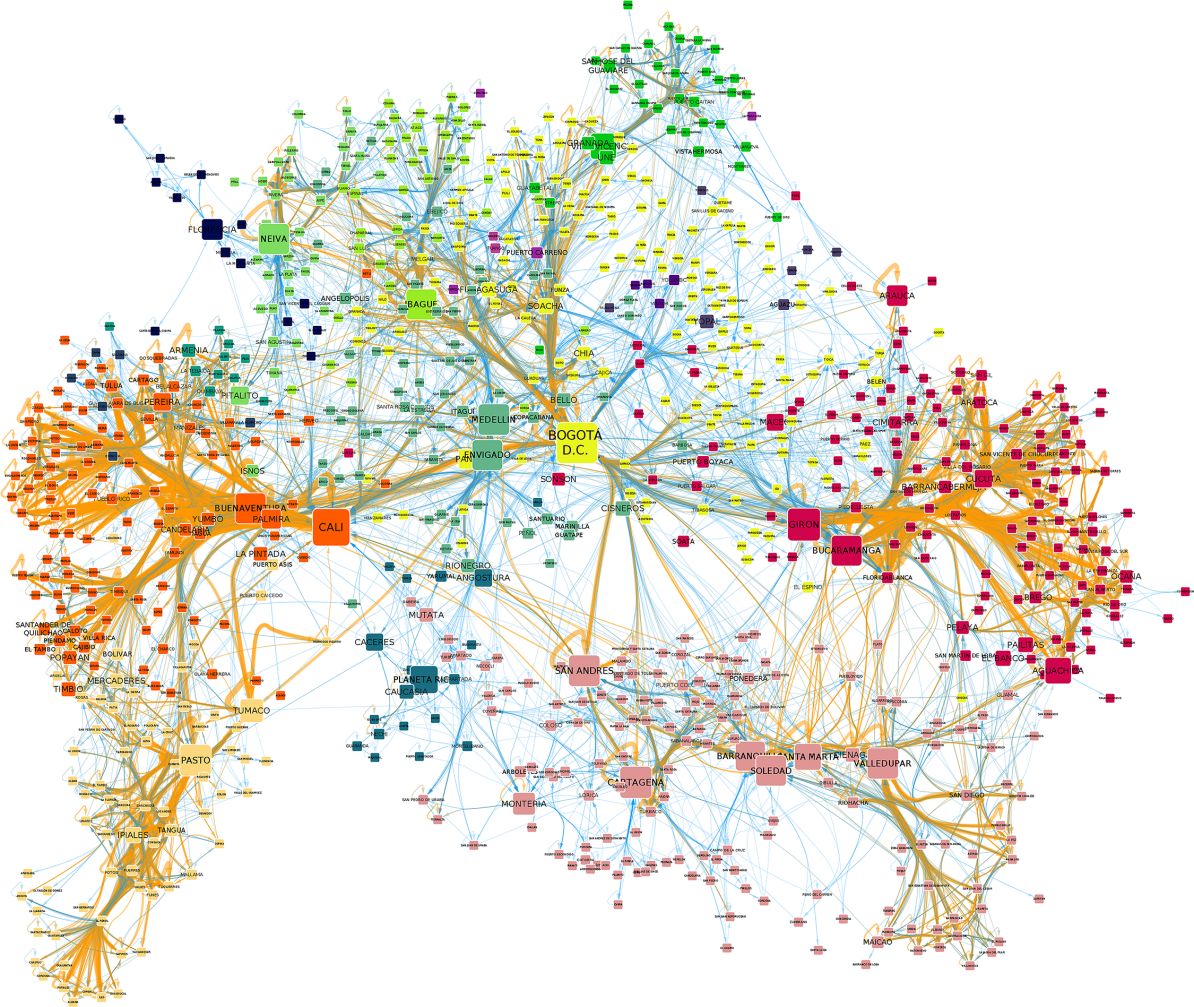
The call-based network of Colombia. The graph representing the call relationships in Colombia across municipalities. Each node is a municipality and directed links connect two municipalities if people from one municipality have a significant amount of call relations with people living in the other municipality. Node size is proportional to indegree, and node color indicates the node community, as detected by Infomap. Link size and transparency is proportional to its significance, as is its color: orange links are very significant, blue links are less strong.

**Fig 2:**
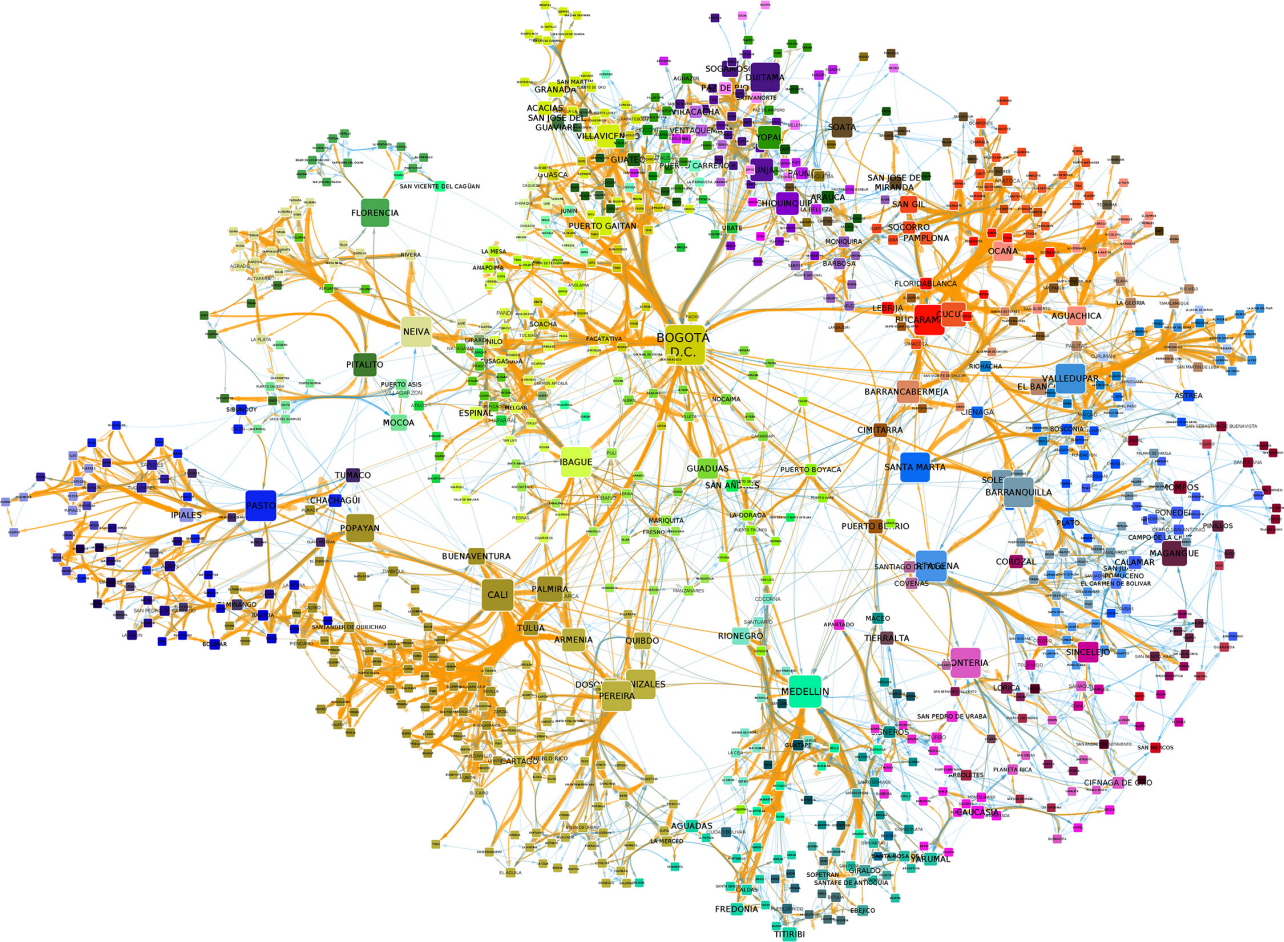
The mobility network of Colombia. The graph representing the mobility relationships in Colombia across municipalities. Each node is a municipality and directed links connect two municipalities if we observe a significant amount of trips flowing from one municipality to another. Graphical elements are defined similarly to the call-based network presented above, and we refer to Fig 1’s caption for their discussion.

Figs 1 and 2 respectively depict the resulting call-based and mobility networks. We calculate some topological features of both networks and report the results in Table 1. We expect to find a strong effect of distance on the mobility network and to find smaller effects on the phone calls network. After all, mobility requires significant energy and time to move our bodies across the country, whether by road or plane. Phone calls, on the other hand, enable people to talk instantaneously with anybody in the country and costs are not affected by distance. We find that calls decay less strongly with distance but they still conform to compact topological features.

**Table 1:** Network statistics of the call-based and mobility networks. We calculate the following topological features of the call and mobility networks: number of nodes (“# Nodes” or *n*), number of edges (“# Edges” or *e*), average degree (“Avg Degree”, $\frac{2e}{n}$), average path length (“Avg Path Length”, the number of edges needed to be crossed to go from a random node of the network to another), degree assortativity (“Degree Assort.”, correlation coefficient of the degrees of nodes connected by an edge), reciprocity (fraction of directed edges going in both directions), and codelength (number of bits required to encode the network given the communities calculated by Infomap, the lower the more well-separated are the communities).

Measure	Social	Mobility
# Nodes	863	863
# Edges	9,639	6,614
Avg Degree	22.34	15.33
Avg Path Length	2.88	4.46
Degree Assort.	-0.142	0.004
Reciprocity	19.87%	37.30%
Codelength	5.156	4.151

The networks are built selecting the most significant edges, using a threshold which can be interpreted as the p-value of the considered connections (for a full explanation about why this is the case, we refer to the original paper proposing this thresholding technique [37]). In the calls-based network we impose a much stricter significance threshold (0.0001, while the mobility threshold equals to 0.01). Even with a much stricter significance threshold, we still obtain more call edges (9,639) than mobility edges (6,614). Note that the different thresholds are chosen in order to minimize the amount of noisy edges. Since there are more candidate edges in the call network, we need to impose a stricter threshold.

**Fig 3:**
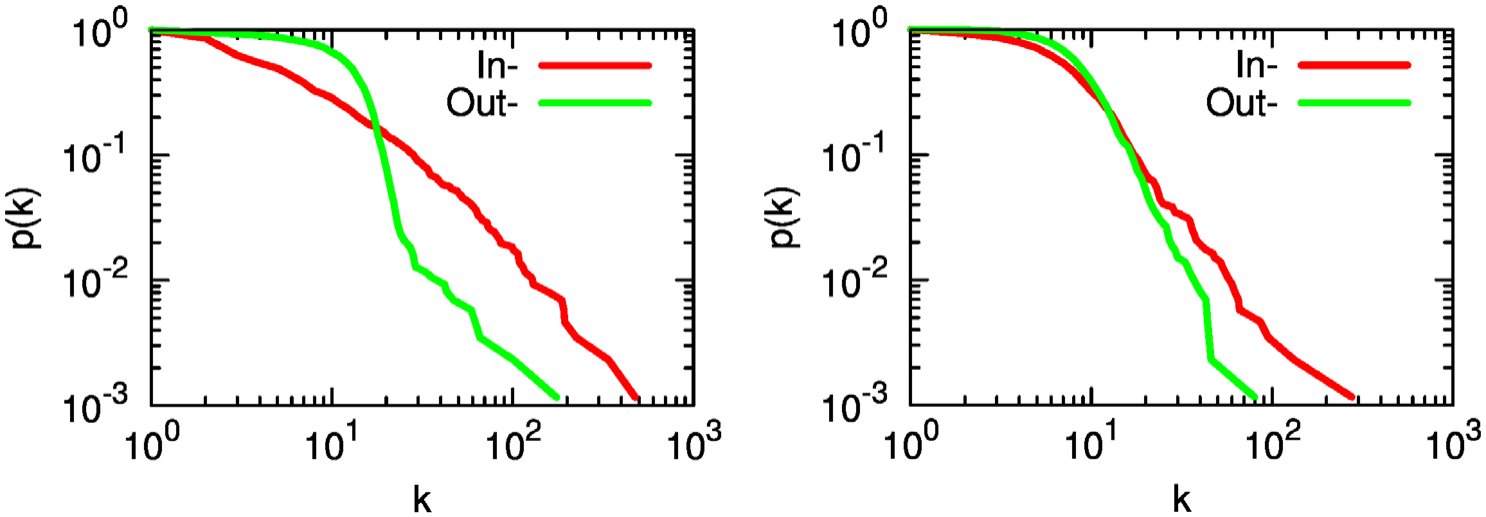
The call-based (left) and mobility (right) degree distributions. Cumulative indegree (red) and outdegree (green) distributions for the call and mobility networks. The plots report the probability that a node in each network has a given degree, or higher. For instance, in the call network there is roughly a 1% probability (*p*(*k*) = 10^−2^) that a node has a indegree of 100 (*k* = 10^2^) or more.

There is a pronounced degree disassortativity and lower reciprocity in the call-based network. Degree disassortativity means that low degree nodes tend to connect to high degree nodes, in a hub-and-spoke logic. These hubs, in turn, do not reciprocate connections, thus there are low levels of bidirectional links. These facts suggest that there is a large difference between in and out degree in the calls network. In fact, the in and out degree distributions in Fig 3 show that they are very different in the calls network, while the distributions vastly overlap in the mobility network. These facts point to the existence of social aggregators: the in-degree distribution looks scale free, while the out-degree does not, and this kind of differential scaling has been observed in directed social networks. It is usually interpreted as a limited bandwidth effect: the number of people a person can follow is bounded, but in principle a single superstar could be followed by everybody [34, 35]. On the other hand, the mobility network has only a few medium scale hubs (e.g. big cities like Bogota and Medellin). Neither the in- nor the out- degree distributions in the mobility network are scale free and they overlap to a larger extent. Another hint that calls span more freely across the territory is given by the average path length in the network, which is lower in the calls-based network (Table 1).

The analysis of simple topological properties of the calls-based and mobility networks seems to suggest a significant difference between them. We now turn to a more advanced analysis of network clusters which aims to show the common properties of the two structures.

### Network Clusters

While the cost of a national phone call does not change with distance, the network based on calls does decay with distance, thus exhibiting spatial clustering. For this paper we define network clusters (or communities) as a set of nodes that are densely connected to each other and sparsely connected with the rest of the network. Network clusters in the calls-based network are one measure of the intensity of social interactions between municipalities. The mobility-based network uncovers relationships that require face-to-face interaction. The algorithm used to detect clusters is Infomap [38] and it calculates the optimal number of clusters to minimize the codelength of the representation. Codelength is an information theoretic concept: it calculates the number of bits required to encode all nodes in the network, given their affiliation to the identified communities. Higher codelength means that more bits are required, implying that separating nodes into a few clusters is not enough because clusters are not cleanly separated. Note that codelength is dependent on the number of nodes in the network: more nodes require more bits to be encoded. But since in our case the two networks have the same number of nodes, the comparison of the codelengths is meaningful. Table 1 reports the codelengths for both networks, showing that, as expected, the calls network has a higher codelength. Comparing the two networks, it is clear that the calls-based clusters are larger in the sense that they include more nodes than the mobility clusters (in Figs 1 and 2 nodes are color-coded depending on the community to which they belong). As a consequence, there are fewer phone-call clusters (22) than mobility clusters (81).

We interpret this result as a reflection of the fact that calls are less affected by distance than mobility connections creating a higher degree of interconnectivity across more distant municipalities. A quantitative estimation of this is the codelength measure.

**Fig 4:**
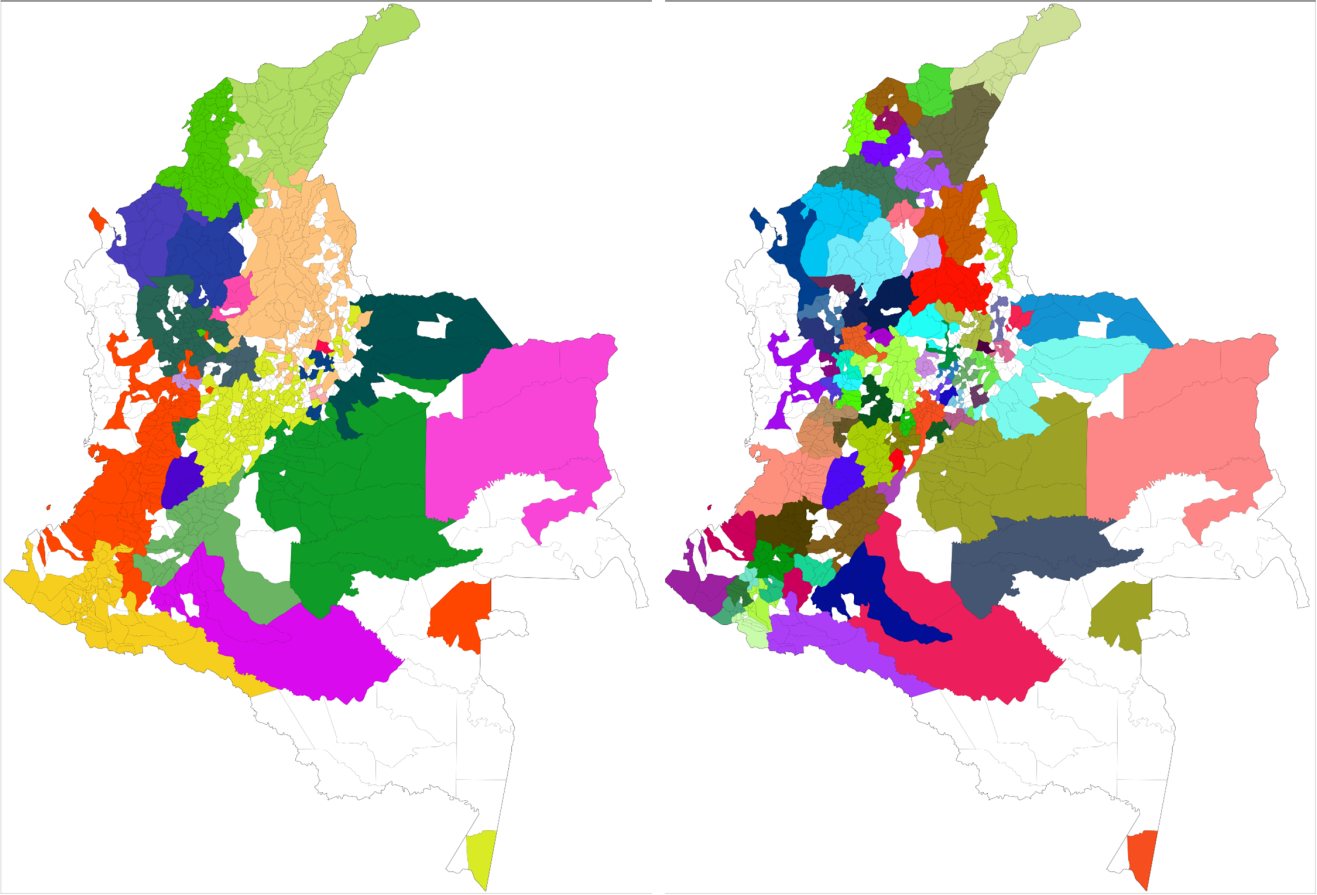
The call-based (left) and mobility (right) clusters on Colombia’s territory. A geographical visualization of the network clusters computed on the call and mobility networks. Each municipality area is colored with its corresponding cluster. The color palette is the same used for Figs 1 and 2, so a node’s color in those figures corresponds to its municipality color in these figures.

**Fig 5:**
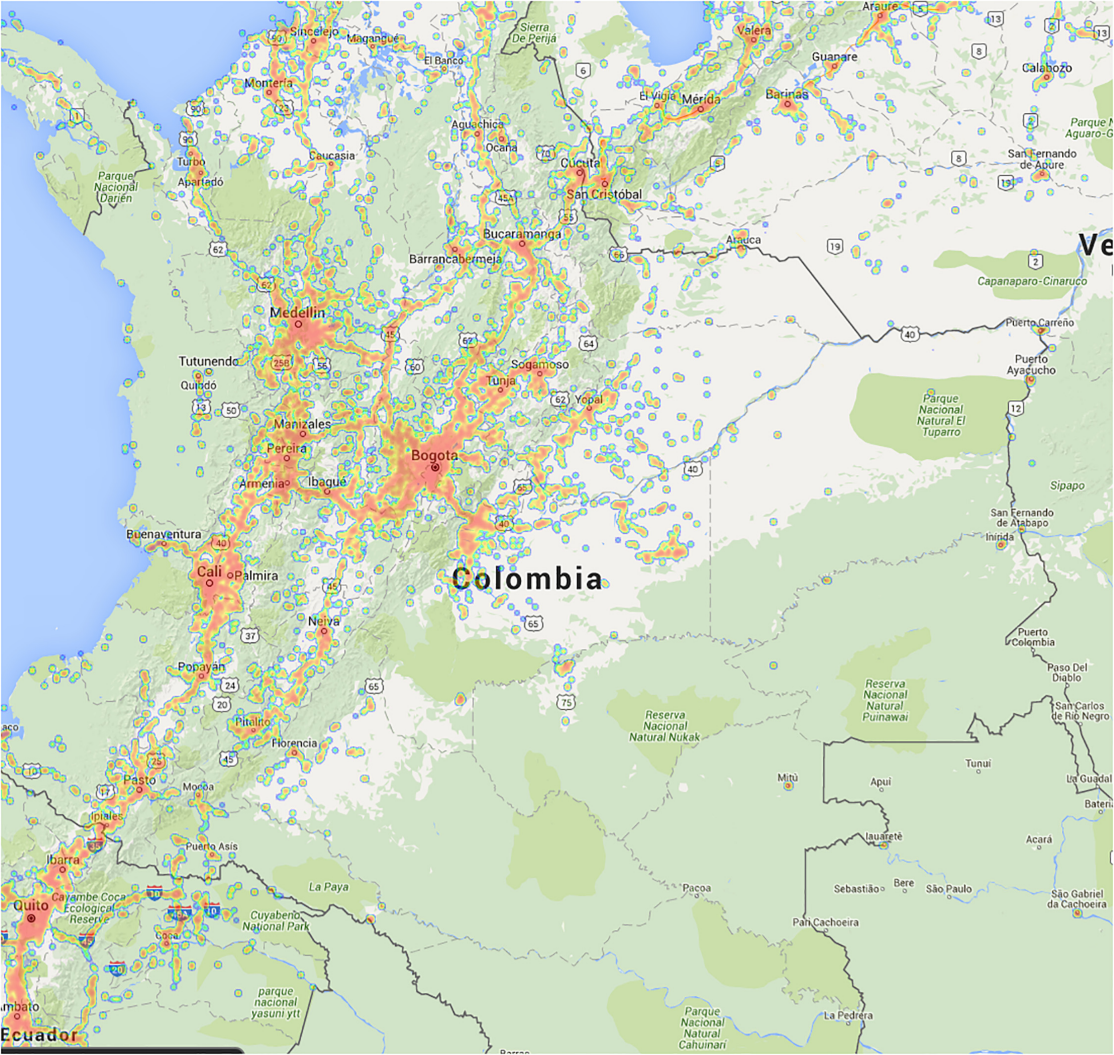
Colombia cellphone signal strength. The heatmap represents the signal strength of the cellphone network across the territory of Colombia. Red areas have a strong signal, blue areas a weak signal, and uncolored map areas have no signal. Image courtesy of opensignal.com, which granted us permission to use it under a CC BY 3.0 license.

In Fig 4 we display the territorial distribution of the calls-based and mobility clusters in Colombia. Note that white municipalities are excluded because they possess no cellphone towers. In these municipalities cellphone connection signal is either very poor or non-existent (see Fig 5). We exclude these areas.

The analysis of the cluster maps in Fig 4 reveals that the calls-based social structure is influenced by geographical distance. If distance did not affect the calls-based social network, its clusters would not be spatially contiguous and we would observe enclaves and long-range structures. Instead, we see that the call-based clusters are highly compact, with very few and small exceptions. This is confirmation of previous works on the relationship between social ties and distance [12, 14].

We extend these previous results by noticing the overlapping relationship between the calls-based social clusters and the mobility clusters. By visual inspection, one can perceive that the smaller mobility clusters appear to be included in the larger call clusters. Visual inspection is confirmed by the overlap analysis. We calculate the degree of overlap of all mobility clusters with all call-based clusters, by counting the fraction of the nodes included in a cluster that are also included in another cluster. If a mobility cluster contains 10 nodes and 9 of them are also included in calls-based social cluster, then the corresponding overlap is equal to .9. We then associate each mobility cluster to a “parent” call cluster, that is the call-based cluster containing most of the child cluster’s nodes. Finally, we estimate the degree of mismatch by counting all nodes that are in a mobility cluster but are not in the corresponding parent call cluster. The observed mismatch is equal to 9.73%, meaning that 9.73% of nodes in the mobility clusters are not present in their parent call cluster.

**Fig 6:**
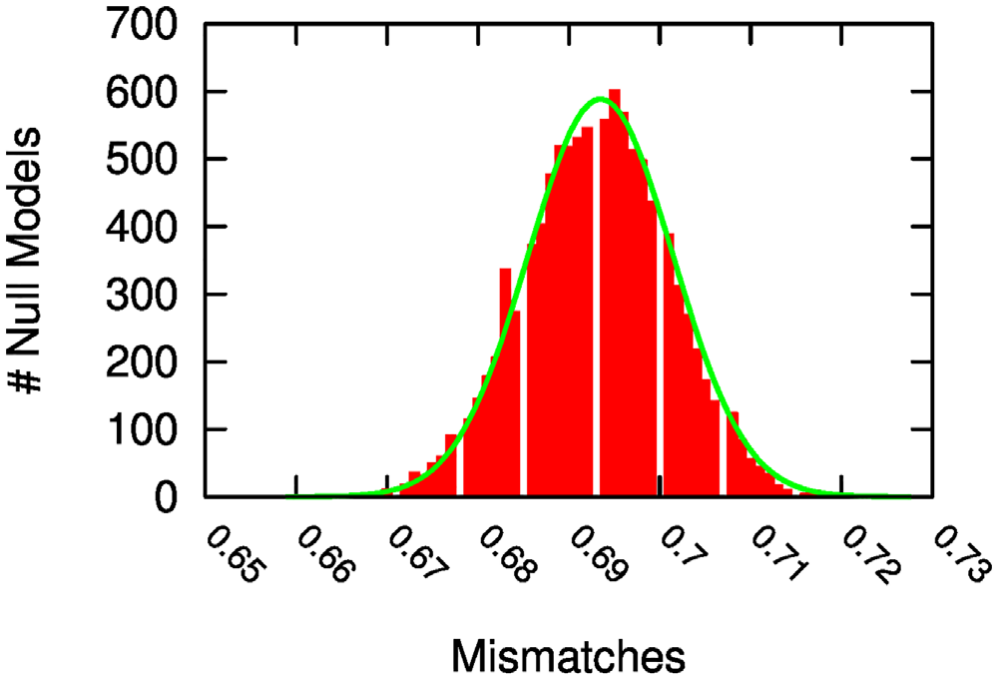
Null model mismatch distribution. We calculate null call-based and mobility community and we test their degree of overlap. The figure reports the number of null models (Y axis) scoring each overlap value interval (X axis). The visible white bands are caused by gaps in the possible overlap values that we can obtain, since the denominator is lower than 1,000 (it is equal to the number of nodes, *n* = 863). Green line fits the most likely probability distribution, which is a Gaussian with *μ* = 0.7287 and *σ* = 0.0074.

We test the significance of this observation through a null model. In the null model we generate 22 random call clusters and 81 random mobility clusters. Each random cluster contains the same number of nodes of its corresponding real-world cluster, but its members are chosen randomly. We calculate the mismatch ratio using the same procedure described above. The average mismatch ratio observed in the null models is around 72%. We ran 10,000 iterations of the null model and Fig 6 reports the resulting mismatch distribution. Given the distribution’s average and standard deviation we can conclude that the observed mismatch ratio carries *p* ∼ 0. We obtain a very comparable expectation and standard deviation from the null model if we randomize only the call-based clusters, keeping the actual mobility clusters fixed.

Note that the result could be explained by noticing that the mobility connections are a subset of the call connections. While this is true (80% of mobility edges are also in the call-based network), it is not a complete explanation of our results. The mobility edges need to be a very specific subset of the call edges. In fact, if we draw random call edge subsets of equal size to the mobility edges, we do not obtain comparable mobility clusters.

Our interpretation of these results is that there is a significant overlap between the calls-based network and the mobility network in Colombia but calls-based relationships are less influenced by distance than relationships that involve mobility. This can be shown by creating a rescaled version of the calls-based network where we rescale the weights by the expected distance-based decay of the mobility network. Given two municipalities *m*_1_ and *m*_2_, *S*(*m*_1_,*m*_2_) and *M*(*m*_1_,*m*_2_) are the weights of their connections in the call-based and mobility networks, respectively; and *δ*(*m*_1_,*m*_2_) is their spatial distance. We can rescale *S* as follows:

$$\begin{array}{c} S^{\prime }\left(m_{1},m_{2}\right)=S\left(m_{1},m_{2}\right)\times f\left(\delta \left(m_{1},m_{2}\right)\right), \end{array}$$

where *f*(*δ*(*m*_1_,*m*_2_)) is the expected mismatch between call and mobility links at the given distance. The details on the formulation of *f* are reported in the Methods section. In practice this equation normalizes the weights in *S*′ by scaling *S* weights as if distance affected them as much as it affects *M* weights. *S*′ is what *S* would look like if phone calls were affected by distance as much as mobility (*M*).

If mobility clusters are really distance-bounded calls clusters, then *S*′ clusters should match perfectly with *M* clusters. We calculate *S*′ clusters following the same procedure used for *M* clusters. We obtain 90 clusters, which is closer to the number of *M* clusters (81) than to the number of *S* clusters (22). We compare the *S*′ clusters with *M* clusters by calculating the Normalized Mutual Information (NMI), which is a standard way to compare partitions. NMI evaluates the agreement of two partitions and it equals 1 for perfectly aligned clusters and 0 for random clustering. In this test, NMI equals 0.89. This remarkable matching between the partitions confirms that rescaling call connections to take into account the effect of distance generates a structure comparable to the one observed by tracking human mobility. This confirms the hierarchical relationship between calls-based and mobility ties. Calls-based clusters can be considered the parents of several mobility clusters. Telecommunications does allow for spatially more extended connections than face-to-face interactions by reducing the impact of distance. However, this impact is attenuated, not eliminated. As a consequence, the calls-based network appears as a higher-order aggregation of the mobility network.

To check that our results are not caused by a particular feature of our community discovery algorithm but by the data itself, we apply our algorithm to a different dataset. In particular, as mentioned above, Duranton identified spatial modules using mobility data based on commuting data [32]. We consider the subdivision of the Colombian territory made by Duranton. This study detected shorter-range clusters, with only 170 municipalities belonging to clusters larger than one municipality. These Duranton clusters are almost completely included in our mobility clusters. We assigned each Duranton cluster to a parent mobility cluster with the same logic we applied before for finding the parent phone-call cluster of a mobility cluster. In this case, we found a mismatch for only seven municipalities out of 170. This means that our mobility clusters are a hierarchical level above the Duranton clusters and they almost completely include them.

We validate our results against two possible edge thresholding criticisms. First: to build the call-based and mobility networks of Colombia, we had to filter out many connections between cities, with a harsh threshold. We did so because the sparser networks are less noisy and easier to visually inspect and interpret. We check whether our results are influenced by this choice, and whether our results would be very different if we used the entire set of connections with no thresholding. We reject this criticism by calculating phone-call and mobility clusters on the full set of connections. We cannot use a community discovery algorithm, because the un-thresholded networks are almost fully connected. Thus, we run the matrix clustering algorithm k-Means instead. We fix *k* to 22 and 81 for the call-based and mobility network, respectively. To compare the agreement between matrix and network clusters we use NMI scores, as done previously for *S*′ clusters. For the call and mobility clusters we obtained NMI values equal to 0.66 and 0.81, respectively. Given that we obtain a strong alignment for the filtered and non-filtered networks, we can conclude that thresholding did not significantly impact our results.

The second criticism involves a multiple hypothesis testing argument. When selecting edges with *p* < 0.01, we are ensuring that the expected number of noisy edges is 1%. However, we could have been stricter and apply a Holm-Bonferroni correction [39] to ensure that the probability of having one noisy edge is 1%. We show that both strategies yield comparable results. We create both a call-based and a mobility network using the Holm-Bonferroni correction fixing the actual p-value at 0.01, for both networks. We then calculate the network clusters of these robust networks and we compare them with the network clusters we discussed so far. We obtain a NMI score of 0.86 for the call-based network and of 0.95 for the mobility network. This proves that our threshold choice did not have a significant influence on the results. In particular, the reason lies in the fact that the Infomap algorithm is designed to handle a certain amount of noisy edges, as many analytic techniques dealing with complex networks are.

Note that the first criticism stated that our threshold was too strict, while the second proposed that it was not strict enough. By addressing both criticisms, we showed the full independence of our results from the threshold choice.

## Discussion

In this paper we analyzed the calls-based and mobility networks in Colombia. The calls-based network is obtained by connecting each Colombian municipality with another municipality if we observe a significant number of phone calls flowing between them. The mobility network is built by observing the physical flow of phones, as measured by the towers through which they connect. We find that the phone-call based network has a structure that is isomorphic to that of the mobility network with connections that decay less strongly with distance.

We confirm that, as with many other facets of social and economic life, the frequency of phone-call and travel decay with distance. Our novel contribution is to show the relationship between the phone-call and mobility borders: mobility borders encompass smaller areas and they are mostly included in a parent phone-call border. This is evidence of the fact that the social relations that are expressed through phone-calls occur in a space that is a higher-order aggregation of the face-to-face relations. Further, we rescale the phone-call network, so that connections decay as strongly as the mobility network. This operation also reveals the very strong similarity of the two structures, expressed in the fact that we obtain the same network clusters.

We consider these results as highly suggestive. The hierarchical relationship between the phone-call and the mobility networks, and their postulated identity, is worth further investigation. In particular, for this paper we limited ourselves to establishing it. Future work could explore the causal mechanism behind this similarity. Which social relations require face-to-face interactions and which can be carried through telecommunications? To what degree are these two forms of communication complements and to what degree are they substitutes? Do telecommunications-based relationships decay over time unless reinforced by lower-frequency face-to-face relations? Could we envision a future where more efficient transportation modes or more realistic telecommunication devices would make social and mobility clusters equivalent?

These networks can also be used to explore the diffusion of different forms of economic and social behavior and activity over space. What are the phenomena that diffuse through face-to-face connections vis-a-vis those that can tolerate longer distance interactions through telecommunications? What are the likely effects of improvements in physical connectivity vis-a-vis telecommunications in the diffusion of the different social phenomena of interest in different areas?

## Materials and Methods

This paper is based on data obtained from telecommunications operators in Colombia. The data includes the metadata of all phone call records initiated by a cellphone card issued by one of these operators in Colombia. For each phone call, we have the following information:

- Encrypted and anonymized ID of the caller, consistent over the dataset (the same ID is always associated to the same cellphone card);
- Encrypted and anonymized ID of the callee, again consistent over the dataset. Note that while source phone numbers are all part of the operators networks, target phone numbers can be from any company and even from outside the country;
- Date and time of the call: the moment in time when the call started. The granularity of the data is at the second, for instance one call started on December 1st, 2013 at 6:07:41 PM;
- ID of phone tower: to which phone tower the source cellphone connected to initiate the call. This ID can be crossed with metadata we have about the cellphone tower to pinpoint the location of the caller when he initiated the call;
- Length of call: how many seconds the call lasted. We drop this information as it is of no interest for this study.

Note that the encrypted and anonymized phone numbers are provided by the telecommunication operators such that it is impossible to identify any individual.

We are unable to share the raw data used for this study for two reasons: there are privacy concerns of potentially identifiable individuals and the complete dataset is of prohibitive size (∼250GB). However, we can share the minimum data set necessary to replicate the analysis (which has been included as S3 File).

From our data, we see that the operators which provided the data clear on average approximately several million calls every day, with a clear weekly pattern. The market is pretty stable, without noticeable long term variations. As we already reported in Fig 5, cellphone coverage in Colombia is very inhomogeneous across its municipalities. The number of antennas per municipality has a skewed distribution, with many municipalities having few antennas and larger municipality hosting many antennas.

Both the call-based and the mobility networks discussed in the paper are generated with the same procedure. We keep only IDs which originated and received at least six calls during the observation period. In this way we can drop foreign phones and all special phone numbers that are likely to be not associated with an actual person (e.g. call centers). It is important to note that, after this cleaning phase, we do not follow any individual phone number. The networks are aggregated at the level of the municipality.

In the case of the mobility network, we connect two municipalities if a caller has been observed traveling from one municipality to the other. For each number, we generate the history of the calls it made, that is a time sequence of municipalities it connected to. We know the time and space difference between two consecutive calls. To add an edge between two municipalities, the phone number has to have made two consecutive calls in these municipalities.

In the case of the call-based network, we first have to associate a phone number to its base location. This is a solved problem: in [40] authors are able to associate a phone with its home and work location. In our case study, we feel that there is no reason to prefer either the home or the work location for a phone. We should associate a cellphone with the area in which it spends most of its activities. For each phone, we calculate the number of calls initiated during our entire observation period and we associate the phone’s location with the tower to which it connects most frequently. Then, we know which other phones each phone called. We count each call as an edge between the two favorite municipalities of the phones involved.

In both cases, we have a directed weighted tower to tower network. We firstly aggregate this network at the municipality level, merging together all towers that are located in the same municipality, and aggregating their edges accordingly. Given the amount of data, the raw network contains vast amounts of noise, and we end up with an almost complete graph where every municipality connects to every other tower. To select the most significant edges, we firstly calculate the maximum spanning tree of the network (using the Kruskal algorithm), to ensure that all municipalities are part of the main network component. Then, we add edges by applying a technique designed to evaluate the statistical significance of an edge’s observed weight [37]. For each edge, the technique returns a significance score which is equivalent to the p-value of the edge.

To detect network clusters, we rely on the large community discovery literature. We chose the Infomap algorithm [38] for two reasons: it performs well [41] and it returns disjoint clusters. Disjoint clusters are preferred because we do not want to deal with overlapping communities, where municipalities could belong to different clusters. We expect large cities to be across clusters, but we do not think that allowing them to span across communities is going to provide any useful insight for this work. On the other hand, it will make it harder to test the overlap between call and mobility clusters.

**Fig 7:**
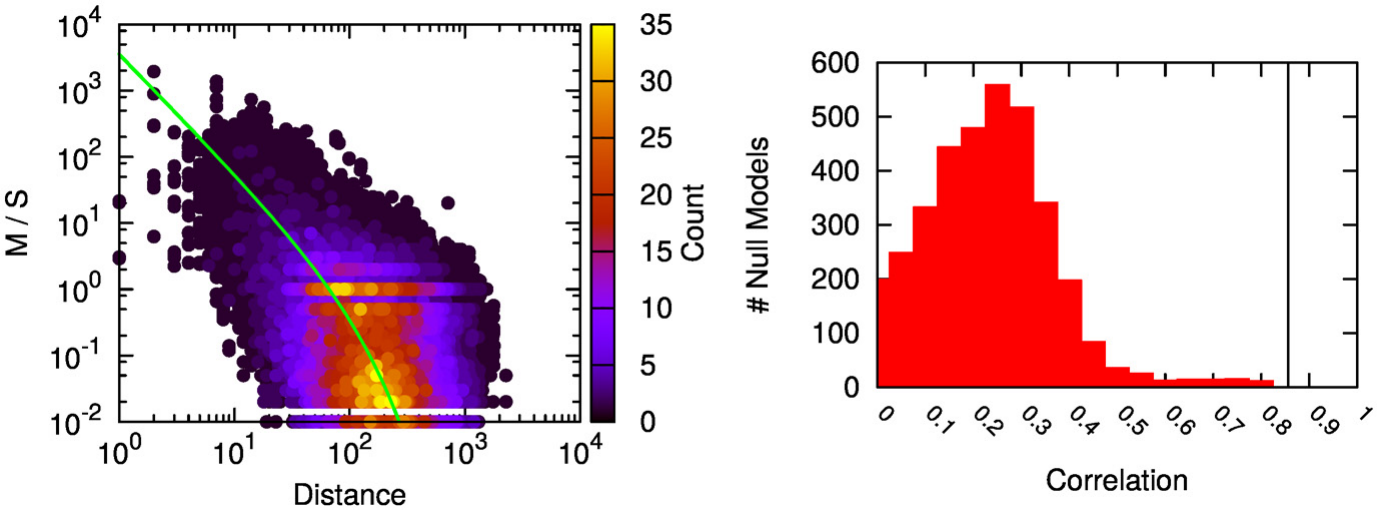
Mobility-Call weight ratio against distance. (Left) In the y axis we report the ratio of the weight of links connecting two municipalities at a given distance (x axis) in the mobility network over the weight of the same link in the call-based network. The green line represents our model function *f*. (Right) Distribution of edge weights correlation coefficients for our distance rescaling null models.

Finally, we need to define the *f* function, whose role is to rescale the weights of the call-based network as if they were affected by geographical distance as the weights of the mobility network. To do so, we need to know the ratio of weights in the mobility network *M* over the weights in the call-based social network *S* at any given distance. We know the distance between each municipality, which is calculated as a straight line between their centers of mass, using the Haversine formula. For instance, if municipalities *m*_1_ and *m*_2_ are 70 kilometers apart, and their mobility and call weights are *M*(*m*_1_,*m*_2_) = 120 and *S*(*m*_1_,*m*_2_) = 90, then we associate a 70 km distance with the ratio $120/90=1.\bar{3}$. When we perform this operation for all observed links, we obtain the scatter plot depicted in Fig 7 (left). The color of the points encodes the number of observations that are associated with the same ratio at the same distance. The best fit found is a truncated power law of the form:

$$\begin{array}{c} f\left(x\right)=\alpha x^{\beta }e^{-x/\gamma }, \end{array}$$

with *α* = 3567, *β* = −1.79, and *γ* = 97. This is the *f* function we use to scale *S* down to *S*′.

Note that *S*′ = *S* × *f*(*x*) and *f*(*x*) = *M*/*S* do not necessarily entail *S*′ = *M*, making our validation circular. If we generate random *S* and *M* weights, keeping the observed distance fixed, and derive their new *f*(*x*) relationship, we do not obtain *S*′ = *M*. If we calculate the correlation coefficient of the edge weights of the randomly generated *M* and *S*′ (as derived from the random *S*), we obtain a distribution of correlation coefficients with average 0.25 and standard deviation of 0.13. Fig 7 (right) depicts the resulting distribution from 3,000 null models. The actual *S*′ and *M* return a correlation score of 0.86 (represented by the black band in the figure).

## Supporting Information

S1 File Image sharing authorization. The document with which OpenSignal granted us the rights of using Fig 5.
(PDF)

S2 File IRB Documentation. The document with which the Committee on the Use of Human Subjects at the authors’ affiliation institution approved the usage of the data made in this paper, certifying that the rights of all subjects whose data have been examined in the study have not been violated.
(PDF)

S3 File Dataset Used. The minimal dataset necessary for the replication of the main results included in the paper. The zip file contains data and code used in the experiments. A readme file provides instructions.
(ZIP)

## References

[1] Bergstrand JH. The gravity equation in international trade: some microeconomic foundations and empirical evidence. The review of economics and statistics. 1985;p. 474–481. 10.2307/1925976

[2] Kleinert J, Toubal F. Gravity for FDI. Review of International Economics. 2010;18(1):1–13. 10.1111/j.1467-9396.2009.00869.x

[3] Jaffe AB, Trajtenberg M, Henderson R. Geographic localization of knowledge spillovers as evidenced by patent citations. National Bureau of Economic Research; 1992.

[4] Branstetter LG. Are knowledge spillovers international or intranational in scope?: Microeconometric evidence from the US and Japan. Journal of International Economics. 2001;53(1):53–79. 10.1016/S0022-1996(00)00068-4

[5] Bottazzi L, Peri G. Innovation and spillovers in regions: Evidence from European patent data. European Economic Review. 2003;47(4):687–710. 10.1016/S0014-2921(02)00307-0

[6] Keller W. Trade and the Transmission of Technology. Journal of Economic growth. 2002;7(1):5–24. 10.1023/A:1013461025733

[7] Keller W, Yeaple SR. Multinational enterprises, international trade, and productivity growth: firm-level evidence from the United States. The Review of Economics and Statistics. 2009;91(4):821–831. 10.1162/rest.91.4.821

[8] Thiemann C, Theis F, Grady D, Brune R, Brockmann D. The structure of borders in a small world. PloS one. 2010;5(11):e15422 10.1371/journal.pone.0015422 21124970 PMC2987795

[9] Ratti C, Sobolevsky S, Calabrese F, Andris C, Reades J, Martino M, et al Redrawing the map of Great Britain from a network of human interactions. PloS one. 2010;5(12):e14248 10.1371/journal.pone.0014248 21170390 PMC2999538

[10] Lambiotte R, Blondel VD, de Kerchove C, Huens E, Prieur C, Smoreda Z, et al Geographical dispersal of mobile communication networks. Physica A: Statistical Mechanics and its Applications. 2008;387(21):5317–5325. 10.1016/j.physa.2008.05.014

[11] Rinzivillo S, Mainardi S, Pezzoni F, Coscia M, Pedreschi D, Giannotti F. Discovering the geographical borders of human mobility. KI-Künstliche Intelligenz. 2012;26(3):253–260. 10.1007/s13218-012-0181-8

[12] Scellato S, Mascolo C, Musolesi M, Latora V. Distance matters: geo-social metrics for online social networks In: Proceedings of the 3rd conference on Online social networks; 2010 p. 8–8.

[13] Takhteyev Y, Gruzd A, Wellman B. Geography of Twitter networks. Social networks. 2012;34(1):73–81. 10.1016/j.socnet.2011.05.006

[14] McPherson M, Smith-Lovin L, Cook JM. Birds of a feather: Homophily in social networks. Annual review of sociology. 2001;p. 415–444. 10.1146/annurev.soc.27.1.415

[15] Cho E, Myers SA, Leskovec J. Friendship and mobility: user movement in location-based social networks In: Proceedings of the 17th ACM SIGKDD international conference on Knowledge discovery and data mining. ACM; 2011 p. 1082–1090.

[16] Wang D, Pedreschi D, Song C, Giannotti F, Barabasi AL. Human mobility, social ties, and link prediction In: Proceedings of the 17th ACM SIGKDD international conference on Knowledge discovery and data mining. ACM; 2011 p. 1100–1108.

[17] De Domenico M, Lima A, Musolesi M. Interdependence and predictability of human mobility and social interactions. Pervasive and Mobile Computing. 2013;9(6):798–807. 10.1016/j.pmcj.2013.07.008

[18] Toole JL, Herrera-Yaqüe C, Schneider CM, González MC. Coupling human mobility and social ties. Journal of The Royal Society Interface. 2015;12(105):20141128 10.1098/rsif.2014.1128 PMC4387518 25716185

[19] Miritello G, Lara R, Moro E. Time allocation in social networks: correlation between social structure and human communication dynamics In: Temporal Networks. Springer; 2013 p. 175–190.

[20] Musolesi M, Mascolo C. Designing mobility models based on social network theory. ACM SIGMOBILE Mobile Computing and Communications Review. 2007;11(3):59–70. 10.1145/1317425.1317433

[21] Noulas A, Scellato S, Lambiotte R, Pontil M, Mascolo C. A tale of many cities: universal patterns in human urban mobility. PloS one. 2012;7(5):e37027 10.1371/journal.pone.0037027 22666339 PMC3362592

[22] Shmueli E, Mazeh I, Radaelli L, Pentland AS, Altshuler Y. Ride Sharing: A Network Perspective In: Social Computing, Behavioral-Cultural Modeling, and Prediction. Springer; 2015 p. 434–439.

[23] Santi P, Resta G, Szell M, Sobolevsky S, Strogatz SH, Ratti C. Quantifying the benefits of vehicle pooling with shareability networks. Proceedings of the National Academy of Sciences. 2014;111(37):13290–13294. 10.1073/pnas.1403657111 PMC4169909 25197046

[24] Bajardi P, Poletto C, Ramasco JJ, Tizzoni M, Colizza V, Vespignani A. Human mobility networks, travel restrictions, and the global spread of 2009 H1N1 pandemic. PloS one. 2011;6(1):e16591 10.1371/journal.pone.0016591 21304943 PMC3031602

[25] Belik V, Geisel T, Brockmann D. Natural human mobility patterns and spatial spread of infectious diseases. Physical Review X. 2011;1(1):011001 10.1103/PhysRevX.1.011001

[26] Halloran ME, Vespignani A, Bharti N, Feldstein LR, Alexander K, Ferrari M, et al Ebola: mobility data. Science (New York, NY). 2014;346(6208):433 10.1126/science.346.6208.433-a PMC4408607 25342792

[27] Schneider CM, Belik V, Couronné T, Smoreda Z, González MC. Unravelling daily human mobility motifs. Journal of The Royal Society Interface. 2013;10(84):20130246 10.1098/rsif.2013.0246 PMC3673164 23658117

[28] Toole JL, Lin YR, Muehlegger E, Shoag D, Gonzalez MC, Lazer D. Tracking Employment Shocks Using Mobile Phone Data. arXiv preprint arXiv:150506791 2015;. 10.1098/rsif.2015.0185 PMC4590504 26018965

[29] Llorente A, Cebrian M, Moro E, et al Social media fingerprints of unemployment. arXiv preprint arXiv:14113140 2014;. 10.1371/journal.pone.0128692 PMC4447438 26020628

[30] Pennacchioli D, Coscia M, Rinzivillo S, Pedreschi D, Giannotti F. Explaining the product range effect in purchase data In: Big Data, 2013 IEEE International Conference on. IEEE; 2013 p. 648–656.

[31] Amini A, Kung K, Kang C, Sobolevsky S, Ratti C. The impact of social segregation on human mobility in developing and industrialized regions. EPJ Data Science. 2014;3(1):1–20. 10.1140/epjds31

[32] Duranton G. Delineating metropolitan areas: Measuring spatial labour market networks through commuting patterns. Processed, Wharton School, University of Pennsylvania 2013;.

[33] Berry BJ, Allen P, et al. Central place studies. A bibliography of theory and applications. Central place studies A bibliography of theory and applications. 1961;.

[34] Szell M, Lambiotte R, Thurner S. Multirelational organization of large-scale social networks in an online world. Proceedings of the National Academy of Sciences. 2010;107(31):13636–13641. 10.1073/pnas.1004008107 PMC2922277 20643965

[35] Kwak H, Lee C, Park H, Moon S. What is Twitter, a social network or a news media? In: Proceedings of the 19th international conference on World wide web. ACM; 2010 p. 591–600.

[36] Cha M, Mislove A, Gummadi KP. A measurement-driven analysis of information propagation in the flickr social network In: Proceedings of the 18th international conference on World wide web. ACM; 2009 p. 721–730.

[37] Serrano MÁ, Boguñá M, Vespignani A. Extracting the multiscale backbone of complex weighted networks. Proceedings of the National Academy of Sciences. 2009;106(16):6483–6488. 10.1073/pnas.0808904106 PMC2672499 19357301

[38] Rosvall M, Bergstrom CT. Multilevel compression of random walks on networks reveals hierarchical organization in large integrated systems. PloS one. 2011;6(4):e18209 10.1371/journal.pone.0018209 21494658 PMC3072965

[39] Holm S. A simple sequentially rejective multiple test procedure. Scandinavian journal of statistics. 1979;p. 65–70.

[40] Toole JL, Colak S, Alhasoun F, Evsukoff A, Gonzalez MC. The path most travelled: Mining road usage patterns from massive call data. arXiv preprint arXiv:14030636 2014;.

[41] Coscia M, Giannotti F, Pedreschi D. A classification for community discovery methods in complex networks. Statistical Analysis and Data Mining: The ASA Data Science Journal. 2011;4(5):512–546. 10.1002/sam.10133

