# Supplementary material for: Evidence That Calls-Based and Mobility Networks Are Isomorphic
Source: PLoS One. 2015 Dec 29;10(12):e0145091. doi: 10.1371/journal.pone.0145091 (PMC4695092; doi:10.1371/journal.pone.0145091)
Supplement: S1 File — The document with which OpenSignal granted us the rights of using Fig 5. (PDF) [file pone.0145091.s001.pdf]

Date: April 27, 2015

OpenSignal
155-157 Farringdon Road,
London EC1R 3AD
+44 (0) 845 834-0987

james@opensignal.com

Dear Sir or Madam,

We authorise the author(s) of the paper to use our figure for publication under the CC-BY or CC0 license, whichever is deemed the most appropriate by the author(s) and/or the editor(s).

Regards,

James Robinson

CTO OpenSignal

OpenSignal · 155-157 Farringdon Road, London EC1R 3AD · Phone: +44 (0) 845 834-0987· Fax: +44 (0) 845 834-0987
