# Supplementary material for: Evidence That Calls-Based and Mobility Networks Are Isomorphic
Source: PLoS One. 2015 Dec 29;10(12):e0145091. doi: 10.1371/journal.pone.0145091 (PMC4695092; doi:10.1371/journal.pone.0145091)
Supplement: S2 File — The document with which the Committee on the Use of Human Subjects at the authors’ affiliation institution approved the usage of the data made in this paper, certifying that the rights of all subjects whose data have been examined in the study have not been violated. (PDF) [file pone.0145091.s002.pdf]

HARVARD
Human Research Protection Program

Harvard University-Area
Committee on the Use of Human Subjects
1414 Massachusetts Avenue, 2nd Floor
Cambridge, MA 02138
Federal Wide Assurance FWA00004837

Notification of Initial Study Approval

February 24, 2015

Ricardo Hausmann
ricardo_hausmann@harvard.edu

**Protocol Title:**	Better understanding how the length and the monetary cost of commuting affect the economic prospects of populations housed in urban peripheries
**Protocol #:**	IRB15-0325
**Funding Source:**	None
**IRB Review Date:**	2/24/2015
**Effective Date:**	2/24/2015
**Expiration Date:**	2/23/2016
**IRB Review Type:**	Expedited
**IRB Review Action:**	Approved

Dear Ricardo Hausmann:

On 2/24/2015, after review of your Initial Study, the Institutional Review Board (IRB) of the Harvard University-Area has approved the above-referenced submission. **Please note that the approval for this protocol will lapse on 2/23/2016.**

This approval includes the following:

- telefonica_CUHS_Protocol_Template as at 02122015.doc (Protocol Documents) version: 0.01

Additionally, the IRB has reviewed the following documents:
- TISA-Harvard-MIT Collaboration Agrmt FULLY EXECUTED.pdf (Data Use Agreement or Other Agreements) version: 0.01
- Telefonica IT requirements.pdf (Other) version: 0.01

The IRB made the following determinations:
- Special Populations: Children
- Waivers: Waiver/alteration of the consent process,
- Risk Determination: No greater than minimal risk

- Research Information Security Level: The research is classified, using Harvard's Data Security Policy, as Level 3 Data.

Please contact me at cuhs@fas.harvard.edu, or 617-496-2847 if you have any questions.

Sincerely,

Jeanne Freeman
FAS IRB
