# Supplementary figures and images for: Evidence That Calls-Based and Mobility Networks Are Isomorphic
Source: PLoS One. 2015 Dec 29;10(12):e0145091. doi: 10.1371/journal.pone.0145091 (PMC4695092; doi:10.1371/journal.pone.0145091)

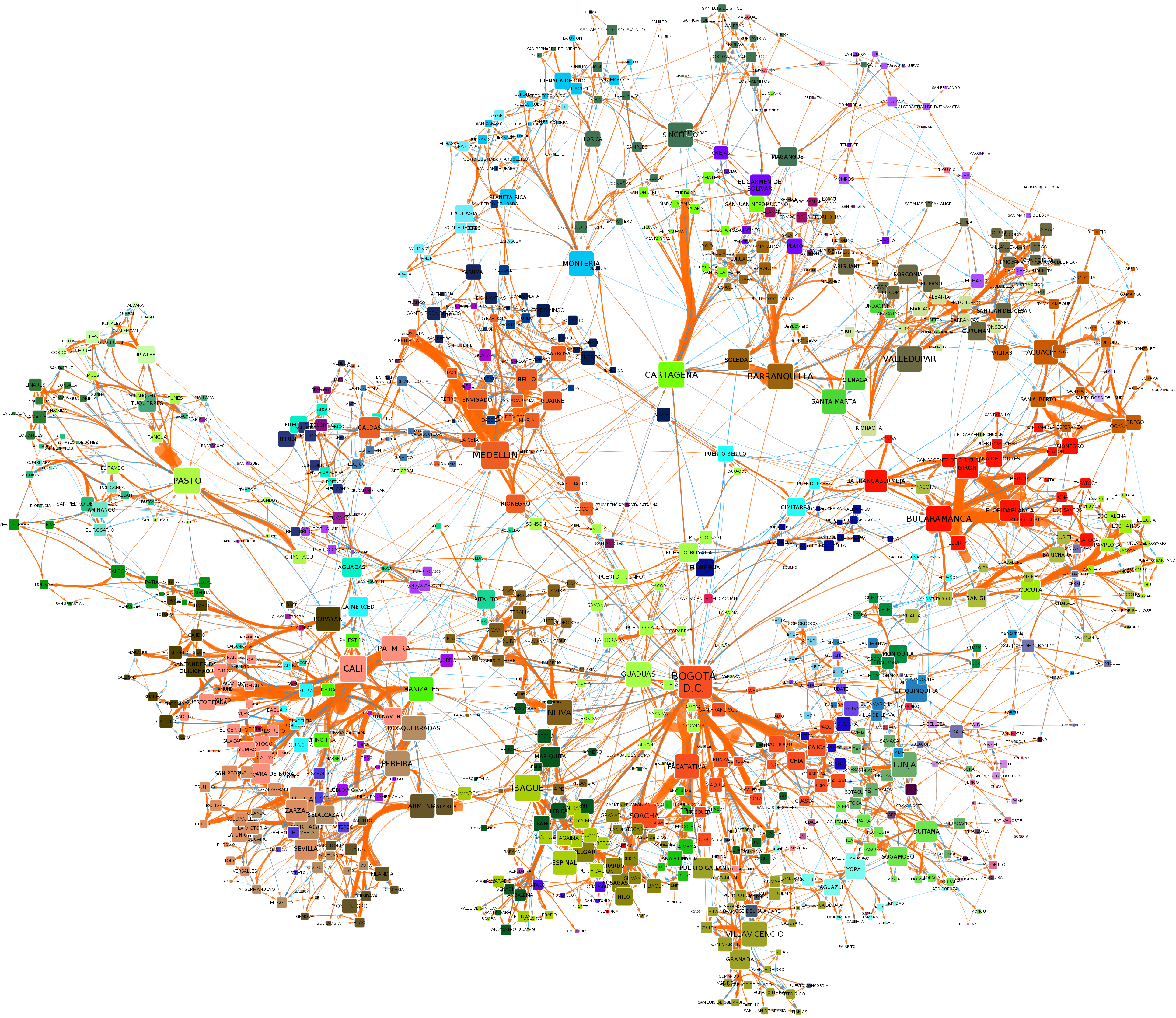

Supplement: S3 File — The minimal dataset necessary for the replication of the main results included in the paper. The zip file contains data and code used in the experiments. A readme file provides instructions. (ZIP) [file pone.0145091.s003.zip › mobility/colombia_mobility.png]

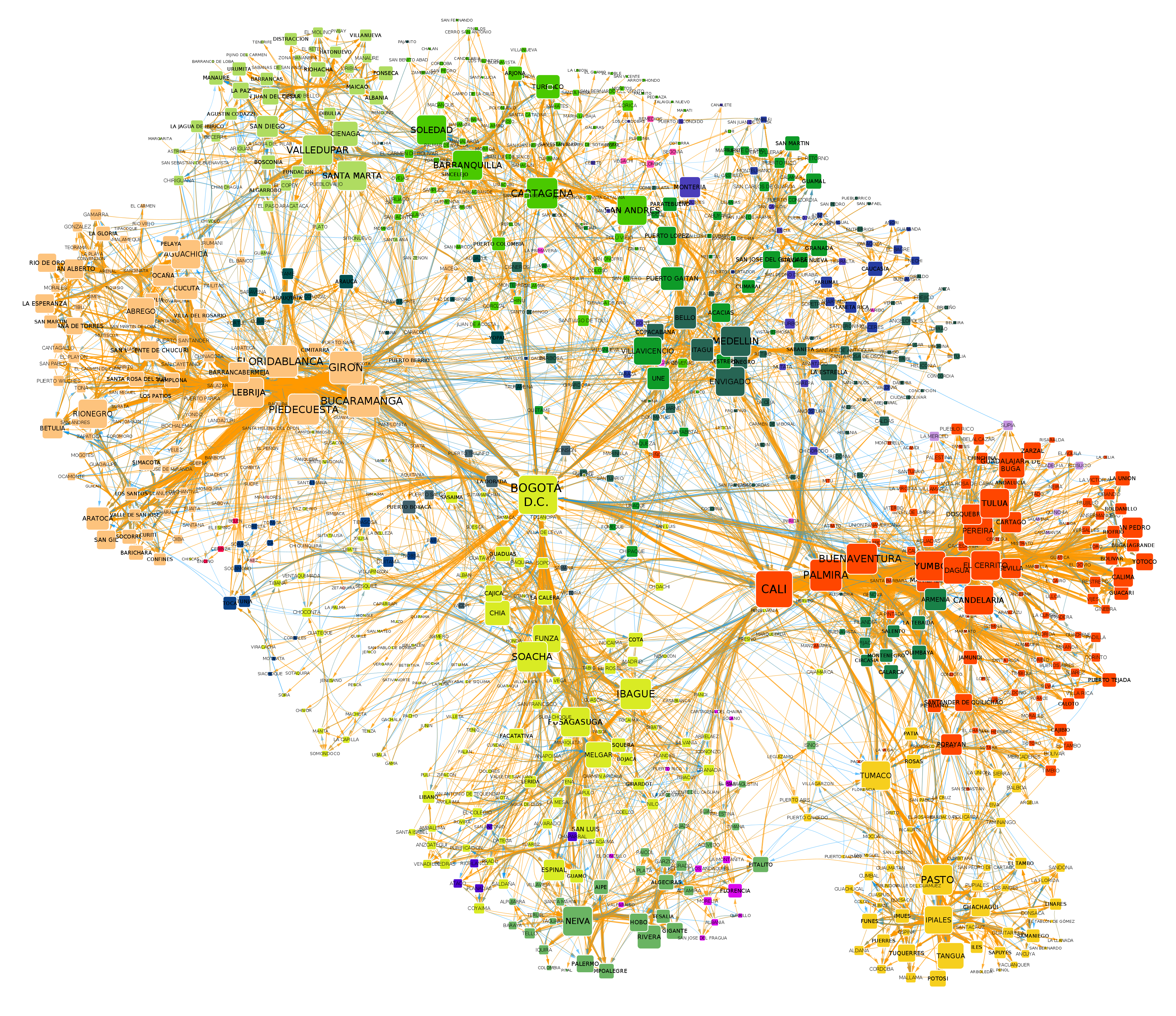

Supplement: S3 File — The minimal dataset necessary for the replication of the main results included in the paper. The zip file contains data and code used in the experiments. A readme file provides instructions. (ZIP) [file pone.0145091.s003.zip › social/colombia_social.png]
